# Exploring the Moderating Effect of Musculoskeletal Pain on Technostress-Induced Burnout: A Cross-Sectional Study of Bank Employees

**DOI:** 10.3390/healthcare12202064

**Published:** 2024-10-17

**Authors:** Seval Kutlutürk Yıkılmaz, Ibrahim Yikilmaz, Mustafa Bekmezci, Lutfi Surucu, Bulent Cetinkaya

**Affiliations:** 1 Department of Physiotherapy and Rehabilitation, Faculty of Hamidiye Health Sciences, University of Health Sciences, Istanbul 34668, Turkey; 2 Department of Management and Organization, Faculty of Business Administration, Kocaeli University, Kocaeli 41380, Turkey; ibrahimyklmz@gmail.com; 3 Department of Defence Study, National Defence University, Ankara 06654, Turkey; mbekmezci@kho.msu.edu.tr; 4 Department of Business Administration, Faculty of Economics, Administrative, and Social Sciences, Bahçesehir Cyprus University, Mersin 10, Nicosia 99010, Turkey; lutfi.surucu@baucyprus.edu.tr; 5 Department of Business Administration, Faculty of Business, European Leadership University, Mersin 10, Famagusta 99500, Turkey; bulent@elu.edu.tr

**Keywords:** technostress, burnout, musculoskeletal disorders, techno-invasion, techno-overload

## Abstract

Background/Objectives: Information and Communications Technology (ICT) advancements and high customer expectations are boosting the use of digital transformation and tech tools in business processes in a competitive environment. This trend enhances business effectiveness and efficiency but also introduces technostress as a new workplace stress factor. Technostress, defined as stress induced by using ICT in the workplace, has become increasingly prevalent in modern work environments, especially in sectors such as banking, due to digital transformation. As technology use intensifies, it raises concerns about potential adverse psychological and physiological effects on employees, particularly in relation to burnout. From a physiological perspective, musculoskeletal disorders (MSDs) are quite common among employees who use ICT for extended periods. MSDs can play a significant moderating role in the relationship between technostress and burnout. In this context, this study aimed to examine the moderating role of MSDs in the effect of technostress on burnout. Methods: This quantitative study surveyed a convenience sample of 220 bank employees, drawing on COR theory, the JD-R model, the P-E fit approach, and transactional stress theory. Data were analyzed using Structural Equation Modeling with SmartPLS 4.0 software, enabling examination of relationships between variables derived from these frameworks. Results: The results reveal that technostress increases bank employees’ burnout experience. Additionally, bank employees with MSDs experience higher burnout levels than those without MSDs. Conclusions: The study’s findings provide valuable insights into managing workplace stress, addressing mental health problems, and promoting employee well-being in the digital age. These results have potential implications for academic understanding and practical applications in sustainable management.

## 1. Introduction

Recent technological advances, artificial intelligence, and Industry 4.0 applications are rapidly digitalizing business processes, enabling work to be completed more quickly and effectively. Digital transformation, supported by developments in information and communication technology (ICT), and the proliferation of mobile devices, collaborative software, and computer networks, significantly enhances users’ ability to access information quickly and easily. This technological synergy allows individuals to work from virtually any location, fostering an environment where information and knowledge can be seamlessly shared with colleagues in real time [1]. Especially in the post-COVID-19 period, applications designed for business processes in various sectors that require less contact and human density, smaller office spaces, and the trend of remote working have greatly improved the efficiency and flexibility of the modern workforce. This has enabled more dynamic and responsive collaboration across different professional settings. While the use of digital tools and the accessibility of employees anytime and anywhere create significant positive effects on efficiency and productivity [2], on the other side of the coin, it triggers significant negative effects on employees [3,4,5]. The constant connection of employees to their work environment; instant communication; multitasking; long virtual meetings; communication through email, WhatsApp, and company software; the use of new digital tools; and the lack of information significantly contribute to stress and anxiety. This negative psychological state is known as technostress [2,6,7].

While technological advances are often viewed as a sign of progress in many areas of society, including the workplace, the development of new business practices and methodologies is also considered a crucial aspect of advancement. This is particularly true in the period following the 2008 economic crisis. In an effort to achieve greater efficiency with fewer employees, there has been a rapid increase in the adoption of technological solutions. However, this trend has led to an increased workload for the remaining employees [8]. The onset of the COVID-19 pandemic has further exacerbated this issue, causing technostress to become an even more intense and widespread phenomenon. Technostress, the stress experienced by individuals due to their use of information and communication technologies, has reached unprecedented levels and affects a broader range of workers than ever before [5,9,10,11]. Technostress, a concept first introduced by Craig Brod in 1984, has been examined from various perspectives over the years. The antecedents of technostress are generally categorized into five key concepts, which provide a framework for understanding this modern phenomenon [7,12]. These aspects highlight how digital advancements, while beneficial, can also introduce new challenges in both work and personal environments. Techno-overload refers to situations where information and communication technology (ICT) requires employees to work quickly and for long hours. Techno-invasion describes the accessibility of employees under all conditions through ICT opportunities and the blurring of work–life boundaries. Techno-complexity refers to employees feeling inadequate due to technological developments. Techno-insecurity relates to the pressure of potential job loss, while techno-uncertainty refers to the uncertainty created by technological developments regarding employees’ work and life, as well as the stress resulting from the need for constant change and renewal [7]. Among these elements, techno-overload and techno-invasion have been identified as technostress’s primary components and dimensions in numerous studies. These factors are particularly prominent due to their significant impact on an individual’s well-being. Researchers have consistently highlighted the importance of these two elements in understanding and addressing technostress. Their effects are especially noticeable in situations where an individual’s mental and emotional health are concerned [13,14]. As a result of technological advances and the use of ICT in the workplace, employees have been experiencing increased workplace stress due to their heavier workloads and the blurring of boundaries between their work and personal life.

With the advent of digitalization, rapid changes in business processes, demands, and applications impact banks and their financial sector employees the most, after the telecommunications sector [15,16]. A study exploring the impact of mobile, internet, and ICT adoption on banks in the EU over a broad period indicates that it significantly enhances performance and profitability [17]. However, the speed and increase in the process of adapting to competitive conditions based on FinTech and digitalization cause significant technostress for employees, triggering essential problems for both employees and the organization [18,19,20,21]. At the organizational level, when considering its effects, it can lead to long-term decreases in employee performance, job satisfaction, and work engagement, as well as potential termination of employment [13,22,23,24,25,26,27,28]. Individually, it disrupts employees’ work–life balance and threatens their physical and psychological health [28,29,30]. Technostress, in particular, causes employees to experience burnout [13,22,31]. The theoretical basis of this interaction can be explained in several theories. The person–environment fit theory emphasizes that technostress can occur due to a mismatch between an employee’s abilities and values and the demands and conditions expected within the work environment. On the other hand, the transactional theory of stress explains that an individual can experience stress based on their evaluation of an event or situation and how their available resources can help them cope with it. This experience is influenced by numerous internal and external factors [32,33,34,35,36]. Once again, Job Demands-Resources and Conservation of Resources Theories supports the idea that employees facing constant high job demands, such as heavy workload and ICT-related stress, will deplete their resources. This could lead to negative experiences when resources are not replenished or when an ideal resource is not provided in the workplace [37,38,39]. Technostress refers to the impact of various job demands related to ICT usage on an individual. It consistently emerges as a significant factor affecting an employee’s interaction with the work environment. This source of stress continuously challenges employees. In the banking sector, amid increasing digitalization and competition, the extensive use of ICT in work processes, coupled with high job demands, leads to techno-overload and techno-invasion. This constant accessibility and intervention in work-related processes inevitably lead to employee burnout as they struggle to replenish lost resources. Technostress not only results in individual burnout among bank employees but also engenders negative workplace experiences that threaten the long-term efficiency and productivity gained through digitalization. Furthermore, the burnout experienced by bank employees disrupts their work–family relationships, increases their intention to leave their jobs, and diminishes their job satisfaction, reducing in-role and extra-role performance [40,41,42,43,44]. In this respect, the interaction between technostress and burnout is an issue that requires special attention.

The stress experienced by employees and the lack of timely intervention for mentally ill individuals currently have a significant cost for businesses, overshadowing profit and performance expectations. A report by the European Agency for Safety and Health at Work highlights that employees in more digitalized jobs (such as the banking sector) experience more work-related stress than those in less digitalized jobs. The report also states that digitalization leads to increased workload and decreased autonomy, ultimately resulting in higher employee stress levels [45]. The Work Health Survey gathered opinions from over 5000 employees across 17 U.S. industries. Eighty-five percent of the participants reported experiencing significant job-related stress, leading to poor mental health. Sixty-five percent of them stated that they had difficulty focusing on their work, which they attributed to their work environment [46]. Another study emphasized that work-related stress resulted in up to 120,000 deaths, placing a $190 billion burden on the healthcare system. Highly demanding jobs increased the likelihood of a doctor-diagnosed illness by 35% [47]. The annual cost of stress-related mental illness in the USA is $3.7 trillion, with the finance, information communications, and manufacturing sectors being the most affected [48]. When considering all of these findings together, it is essential to examine the effect of technostress on employee burnout caused by increasing digitalization, particularly in the banking sector. This detailed examination of the mechanism is crucial for a sustainable management approach. Also, in the literature, the link between technostress and burnout remains debated in academic circles [25,49]. It is important to note that there is insufficient empirical evidence regarding the link between technostress and burnout [50]. Conflicting results emerge when the issue is approached from different perspectives, and further studies are required to examine how job stress induced by information and communication technology (ICT), known as technostress, contributes to burnout. In the studies conducted, it is often emphasized that the sample is either too general or too narrow, for example, focusing only on health, education, or IT workers. It is pointed out that the issue should be addressed across different sectors and professions [51,52,53,54]. To address the concerns raised in the literature, the aim is to investigate the relationship between technostress and burnout among banking employees in the banking sector, where digitalization and ICT usage are prevalent. With this objective in mind, the following hypothesis has been formulated to explore the interplay between technostress experiences and the levels of burnout among banking professionals, drawing from the existing literature and theories such as person–environment fit, transactional theory of stress, job resources demands, and conservation of resources theory:

**H1:**  *Technostress perceptions of bank employees affect their burnout levels.*

**Figure 1:**
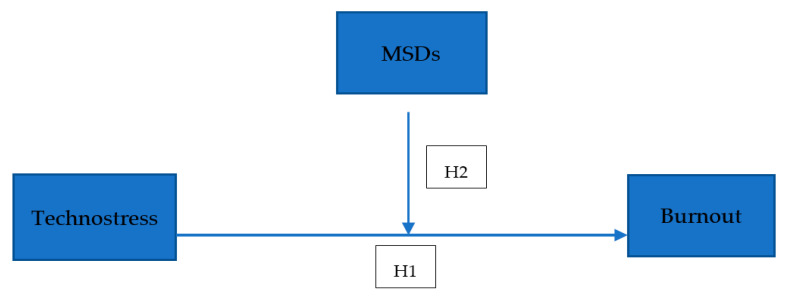
Research Model.

The proliferation of information and communication technologies (desktops, laptops, mobile applications…) has surged in recent years. This increase in usage has correspondingly led to a rise in upper extremity injuries and musculoskeletal disorders (MSD) [55,56]. Increased screen time and repetitive motions, long working hours, improper ergonomic setups, prolonged periods of sitting, and inadequate breaks exacerbate the risk of developing an MSD [57]. Again, as a psychosocial factor, work-related stress (such as technostress) experienced by employees also paves the way for an increase in MSDs [58,59,60,61]. Occupational stress can potentially induce significant changes within the nervous system, altering hormone levels and elevating blood pressure. These physiological responses can increase musculoskeletal co-activation, where muscles contract simultaneously and more intensely than usual [62]. This heightened level of co-activation places additional strain on the musculoskeletal system, which can exacerbate existing musculoskeletal disorders (MSDs) or even contribute to developing new ones [63]. In high-job-demand work environments with technostress, employees’ experience of MSDs increases, and they experience MSDs in different areas, especially in the neck, shoulders, and lower back [63,64,65,66]. MSDs emerge when the demands on the musculoskeletal system surpass its capacity to bear the load [67]. Various studies have identified a connection between prolonged computer use and the onset of musculoskeletal symptoms [68,69,70]. Once again, employees facing long-term ICT-based job demands may experience technostress, leading to burnout. Several factors, including musculoskeletal disorders, contribute to burnout in this context, such as employees’ fatigue levels, chronic diseases, and headaches [71]. Therefore, it is clear that MSDs may moderate the burnout that bank employees experience due to high levels of technostress due to increasing digitalization, technological workload, and techno-invasion experiences. In other words, whether bank employees have MSDs, especially within the digitalized business environment, may make a difference in their burnout experiences. By identifying this relationship, the impact and role of MSDs in the mechanism between technostress and burnout can be revealed, leading to suggestions for measures to be taken by policymakers and senior management. Given the inevitability of technology and digitalization, especially in the banking sector, and the consequent increase in employees’ technostress levels, it is important to identify the variables contributing to the interaction between technostress and burnout. Taking action in this area will be crucial in reducing burnout’s impact on the organization and individuals, and in maintaining a sustainable management approach. Also, the literature emphasizes the need for moderator variables to understand the effects of technostress/ICT-induced stress and to identify mechanisms for reducing these effects. It points out that there are deficiencies in using moderator variables and that the ones used are generally related to technology, such as technical support and literacy facilitation [72,73,74]. There is a call to increase the number of studies on this subject, indicating that the nature of the relationship between stress, burnout, and MSDs is complex. It is suggested that a more understandable interaction mechanism can be revealed within a narrower sample and the scope of specific types of stress [62,75,76]. In this context, to examine the mediating role of MSDs in the effect of technostress, as a particular type of stress, on burnout in the banking sector, within the scope of the extant literature, the following hypothesis has been developed, and the theoretical research model is presented in Figure 1:

**H2:**  *MSDs play a moderating role in the effect of bank employees’ technostress levels on their burnout perceptions.*

Reducing stress in the workplace has significant positive impacts on business outcomes. According to research, alleviating stress can lead to a remarkable 37% increase in sales [77]. Additionally, productivity levels can improve by 31%, allowing employees to accomplish more within the same time frame. Furthermore, the accuracy of task execution is enhanced by 19%, reducing errors and improving overall quality. These statistics highlight the critical importance of fostering a low-stress work environment to boost both efficiency and business performance. In this context, the study aims to create awareness for reducing stress levels in work environments by determining the effect of bank employees’ technostress perceptions on burnout levels and the role of MSDs in this interaction. The empirical findings obtained in accordance with this purpose are discussed within the scope of the extant literature, and critical theoretical and managerial implications are shared. The study’s results are expected to contribute significantly to practice, emphasizing the relationship between technostress and burnout and the role of MSDs in this interaction, thus contributing to the sustainable management approach and employee well-being. Additionally, the study expands the literature by examining the moderator role of MSDs in an empirical framework in the interaction mechanism between technostress and burnout, a new type of work stress when digitalization rapidly conquers every sector.

## 2. Materials and Methods

### 2.1. Research Purpose and Sample

The study’s primary objective is to investigate the impact of technostress on bank employees’ burnout levels and to explore the moderating role of MSDs (musculoskeletal disorders) in this relationship. To achieve this, a cross-sectional research design was implemented. Ethical approval for the study was obtained from the European Leadership University Ethics Committee (01.06.2024/ALU-ETK-2024-07), ensuring adherence to ethical principles and approaches throughout the research process. The study population comprises employees from private and public banks in Istanbul. This city was chosen due to its unique characteristics, including the following:

- Its cosmopolitan structure and advanced technological infrastructure within the Turkish context;
- The diverse range of customers it serves (both commercial and individual);
- The numerous opportunities it presents for comprehensive research.

These factors make Istanbul an ideal location for examining the complex interplay between technostress, burnout, and musculoskeletal disorders among bank employees. The study used a non-probability convenience sampling method called Snowball. This involved asking initial participants to share the questionnaire link with other bank employees to expand our reach. The survey was created and distributed using the Google Forms platform over five weeks between July and August 2024. Participants who agreed to take part in the study and accepted the informed consent form completed the questionnaire voluntarily and anonymously online. Criteria for inclusion were as follows: i. at least one year of work experience in a public or private bank in Istanbul, ii. daily professional use of ICT, and iii. active employment in any position in the bank. A total of 220 participants were reached within the scope of the study.

### 2.2. Measurement Tools

In line with the study’s primary purpose, valid and reliable measurement tools frequently utilized in existing literature were preferred. The study measurement tool consists of demographic questions (such as age, marital status, position, organization type, etc.), as well as questions about technostress, burnout, and MSD variables. Detailed information about the measurement tools related to the main variables in the study is provided below: Technostress: Contemporary research highlights the multifaceted nature of technostress, a phenomenon experienced by employees in the digital era. Scholars have identified five primary components contributing to this modern-day challenge (techno-overload, techno-invasion, techno-complexity, techno-insecurity, and techno-uncertainty). Recent studies propose expanding this framework, suggesting additional factors that influence technostress. These include dependency on technology, lack of fit between demand and ability, flexibility, and communication overload [7,11]. In a study examining the psychosocial reflections of technostress on biological markers and burnout, it was observed that techno-overload had a more significant effect on burnout compared to other components [78]. The techno-overload component is primarily conceptualized as technostress in studies related to burnout [34]. The techno-invasion component is an essential obstacle to employees obtaining the resources they need, and it negatively affects their non-work life in a way that creates stress in the non-work environment. Two sub-dimensions were considered as components of technostress in accordance with the study’s design, which is based on J D-R and COR theories. This approach is a method used in most technostress studies [13]. Once again, in a comprehensive review of studies conducted on technostress between 2008 and 2021, it was highlighted that the components of technostress creators, such as techno-overload (83.3%) and techno-invasion (74.1%), were mainly analyzed in relation to work-related and health-related outcomes [14]. In this context, the items related to techno-overload and techno-invasion, as second-order components of technostress, were extracted from the scale developed by Tarafdar et al. and used to assess the level of technostress experienced by bank employees [7]. Five items were used to measure techno-overload, and four were used to measure techno-invasion dimensions. These items were measured on a 5-point Likert scale ranging from 1 (strongly disagree) to 5 (strongly agree). Example items include “Technology obliges me to work faster” and “Technology obliges me to be permanently in contact with work, even in my free time.” The original English scale was translated into Turkish using the back-translation method. An English-proficient researcher translated the Turkish versions back to English, and researchers refined the Turkish scale to ensure it closely matched the original English versions. Two researchers familiar with the study reviewed the scales for relevance and clarity. Additionally, a pretest with ten employees was conducted to validate the scales further, and based on the results, researchers simplified and improved any ambiguous items to enhance clarity.

The Nordic Musculoskeletal Questionnaire: The Nordic Musculoskeletal Questionnaire, developed by Kuorinka et al., widely used in MSD detection as emphasized by Crawford, and adapted to Turkish by Kahraman et al., was used to measure the MSDs of bank employees [79,80,81]. The Nordic Musculoskeletal Questionnaire (NMQ) comprises 27 questions that assess the occurrence of musculoskeletal issues across nine body regions (neck, shoulders, upper back, elbows, wrists/hands, lower back, hips/thighs, knees, ankles/feet) over 12 months. Additionally, it evaluates symptom severity based on functional impact and recent (7-day) symptom presence. Participants respond to each item using a simple ‘yes’ or ‘no’ format, allowing for straight-forward data collection and analysis of musculoskeletal health status. If participants experienced discomfort in at least one of the nine body regions, the participant was considered to be experiencing an MSD.

Burnout: The burnout experiences of bank employees were assessed using a 4-item burnout scale. This scale has been tested for validity and reliability by Şahan and colleagues. It is the Turkish version of the Copenhagen Psychosocial Questionnaire Version-3 [82]. These items were measured on 5-point Likert scales ranging from 1 (Never) to 5 (Always). Example items are as follows: “How often have you felt worn out during the last four weeks?” and “How often have you been emotionally exhausted during the last four weeks?”

### 2.3. Analyses

The analyses were conducted using Smart PLS 4 and SPSS 27 programs.

#### 2.3.1. Descriptive Analyses

To ensure the study’s viability and applicability, we conducted a thorough descriptive analysis of the participants’ demographic structure. This involved transferring the data to a virtual environment using the SPSS program, identifying key variables, conducting statistical measurements, and visualizing the data. This approach offers a clear overview of participant demographics, which strengthens the study’s reliability and relevance.

#### 2.3.2. Measurement Model Analysis

Before conducting hypothesis tests in the study, analyses were performed to determine whether the existing model and data were suitable for examination within the scope of the structural equation model and whether they met the necessary reliability and validity conditions.

#### 2.3.3. Hypothesis Testing

In the Hypothesis Testing phase, Bootstrap-based regression analysis was first conducted between variables within the scope of structural equation modeling using the Smart PLS 4 program. Then, multi-group analysis (MGA) was carried out to examine the two groups with different MSD levels within the framework of the primary purpose of the study. Hair et al. highlight that multi-group analysis (MGA) in Partial Least Squares Path Modeling (PLSPM) is a highly effective technique for evaluating moderation across various relationships within a model [83]. Traditional moderation analysis typically focuses on a single structural relationship, explicitly examining the interaction effect between an independent variable and a moderator variable on a dependent variable. This involves assessing how the product of the independent variable and the moderator variable influences the dependent variable.

In contrast, MGA provides a broader assessment of the moderator’s influence. Rather than limiting the analysis to one specific relationship, MGA evaluates the moderator’s impact on all relationships within the model. This approach offers a more holistic understanding of how the moderator affects the overall analysis results, giving researchers a deeper insight into the dynamics of models.

According to Hair et al., this comprehensive evaluation makes MGA a robust method for understanding the complexities of moderation in PLSPM, as it allows for the examination of interactions across the entire model rather than in isolation [83,84].

## 3. Results

The evaluation of both measurement and structural models was conducted utilizing Smart PLS 4, an advanced and reliable software for examining variations in exogenous variables. Partial Least Squares (PLS) is a powerful and versatile analytical technique that is widely used for assessing the explanatory power and predictive accuracy of models. By leveraging this method, researchers can gain deeper insights into the relationships and interactions among variables, ensuring that the models provide a comprehensive understanding of the data. This approach not only helps identify significant predictors but also validates the robustness and reliability of the models developed.

### 3.1. Descriptive Statistics

**Figure 2:**
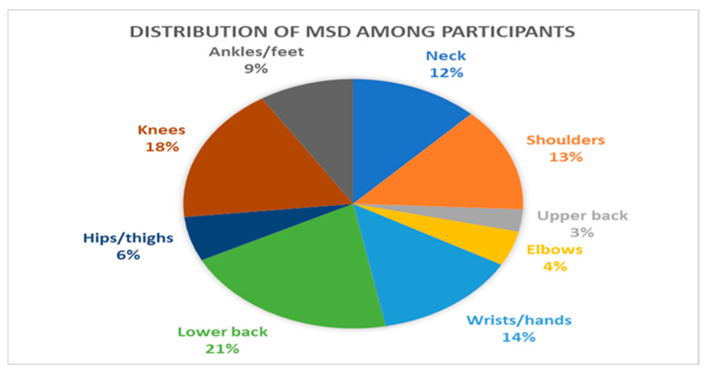
Distribution of MSDs among participants.

In the study, the participants’ average age was 36.5 ± 7.217, with 67.27% being male and 76% being married. When examining the positions of the employees, it is found that approximately 50% of them are at the team-member level. About 51% of the participants work in private banks. The detailed distribution of the complaints related to musculoskeletal disorders (MSDs) experienced by the participants can be found in Figure 2. Overall, it was observed that 54% of the participants experienced pain in at least one area. Analysis of the data on MSDs experienced by employees reveals that the lower back (21%), knees (18%), and shoulders (13%) are the most commonly affected body areas.

### 3.2. Measurement Model Analysis

Following the guidelines provided by Hair et al., the factor loadings of the scale items are examined [85]. Items with loading values below 0.7 were flagged for further review. Specifically, those with loading values lower than 0.40 were immediately removed from the analysis. For items with loading values ranging between 0.40 and 0.70, we evaluated their average variance extracted (AVE) and Composite Reliability (CR) values to ensure they met the acceptable thresholds. In addition, the discriminant validity value is checked to see whether the item below the critical value threatens the validity of the scale. As a consequence of this preliminary scale analysis, one item from the techno-overload scale (tov4-0.397) was excluded from the overall analysis due to its inadequate loading values.

In the second stage, internal consistency was assessed using Cronbach’s alpha, composite reliability (CR), and average variance extracted (AVE) for convergent validity. Following Hair et al., Cronbach’s alpha and CR were examined for construct reliability [84]. As shown in Table 1, both metrics exceed the threshold value of 0.7, indicating reliable scales [86]. Additionally, AVE was calculated to confirm convergent validity. Table 1 shows values (0.576–0.771) above 0.5, meeting the criteria for convergent validity [87].

Discriminant validity was assessed using the Fornell–Larcker criterion, which involves comparing the square root of the average variance extracted (AVE) to the inter-construct correlations. If the AVE surpasses the inter-construct correlation, discriminant validity is established [88]. According to Table 2, the model meets the Fornell–Larcker criterion for discriminant validity. Nevertheless, relying solely on this method may be inadequate, as highlighted by Henseler et al. [89]. Therefore, a Heterotrait–Monotrait ratio (HTMT) analysis is also advised. Table 3 shows that the HTMT values are below the 0.90 threshold, confirming discriminant validity [89].

Finally, following Hair et al., the Variance Inflation Factor (VIF) was assessed to determine the study’s suitability for path analysis [85]. According to the literature, VIF values should be under 10 [90]. Table 1 shows VIF values ranging from 1.527 to 4.049, all below the threshold, indicating no multicollinearity issues. Considering these values, the study model and data are suitable for path analysis.

#### Second-Order Analysis

The study examines technostress experienced by employees, which is a second-order reflective construct consisting of techno-invasion and techno-overload sub-dimensions. The analysis reveals that the data in Table 1 , Table 4, and Table 5 demonstrate the construct’s ability to meet the necessary threshold values in the literature, indicating a valid and reliable structure.

### 3.3. Hypothesis Testing

After confirming that the measurement model meets the necessary reliability and validity requirements, the relationship between the variables was explored using path analysis within the research model. Path analysis was conducted utilizing 5000 resampling boot-strapping in Smart PLS 4 [91]. To assess the significance of the β values derived from the study at a 5% significance level, *t*-tests and *p*-values were scrutinized. According to the hypothesis test outcomes, it was found that technostress had a significant direct effect on burnout (β = 0.413, t = 6.879, *p* < 0.01). The effect of technostress experienced by employees on burnout is approximately 17%. Cohen provided guidelines for evaluating R2 values of endogenous latent variables: 0.26 is considered substantial, 0.13 is moderate, and 0.02 is weak [92]. It is observed that the R2 value of 0.171 obtained from the research indicates a moderate effect. Consequently, the hypothesis H1 was supported.

In the study, a multi-group analysis was conducted to test the main hypothesis that MSDs play a moderator role in the relationship between technostress and burnout experienced by bank employees. Hair et al. state that MGA in PLSPM is highly effective for assessing moderation across multiple relationships [83]. Unlike standard moderation, which looks at the interaction between an independent variable and a moderator variable in a single relationship, MGA evaluates the moderator’s effect on all relationships within the model [83,84]. The results of the MGA conducted within the Smart PLS program are presented in Table 6.

**Figure 3:**
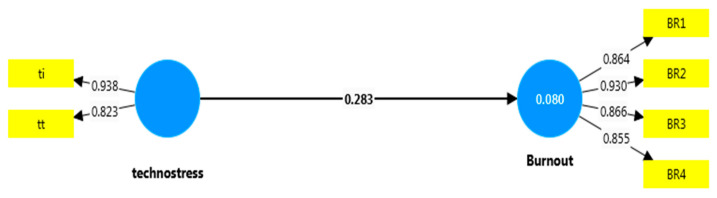
Effect of MSD-0 group on burnout.

**Figure 4:**
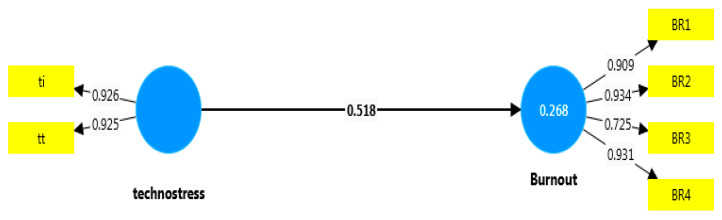
Effect of MSD-1 group on burnout.

When Table 4 is examined in detail, the difference between the path coefficients in the relationship between technostress and burnout is statistically significant. This difference shows that the effect of technostress on burnout experienced by employees with MSDs is stronger than that of employees without an MSD. Additionally, as seen in Figure 3 and Figure 4, the technostress experienced by those with MSDs (MSD-1 group) has an effect of approximately 27% on the burnout they experience. This effect is considered substantial [92]. When the findings obtained in the study were collectively evaluated, it was found that the H2 hypotheses were supported.

## 4. Discussion

This study investigated how technostress experienced by employees in the banking sector affects burnout. Additionally, to better understand this interaction mechanism, musculoskeletal disorders (MSDs) experienced by bank employees were considered as a moderator variable. The results of the study are as follows:

The recent increase in digitalization, mainly due to the impact of the pandemic, has led to significant changes in the way businesses operate and how they are managed, particularly in the finance and telecommunications sectors. The accessibility, flexibility, and practical applications of information and communication technology (ICT) now allow employees to engage with business processes at any time. However, this increased reliance on technology, often referred to as the “dark side,” can lead to technostress among employees. Additionally, the rapid changes in business processes within the banking sector have led to the problem of burnout among employees [93,94]. In the context of pursuing high performance, efficiency, and customer satisfaction, the impact of work-related stress factors such as technostress due to the accessibility, workload, techno-overload, and techno-invasion created by technology is leading to a decreased overall well-being of employees in the medium and long term [95]. This situation not only results in negative experiences for the employee but also decreases performance and employee engagement in the medium and long term [25,96,97]. When examining the theoretical background of this event, the transactional approach views stress as the result of an interaction between the individual and their environment [33,98]. When an individual faces a threat, they assess it and engage in a strategy to manage the situation or alter the conditions [99]. The person–environment fit approach emphasizes the importance of harmony in the interaction between the individual and the environment. Technostress occurs when there is a mismatch in the interaction of the employee with the work environment [100]. The factors that contribute to this mismatch are the reasons that lead the individual to not align with job demands [1,101,102] and can trigger many negative workplace experiences, including decreased employee performance, burnout, and leaving the job [103]. Specifically, the misfit between the employee and the work environment can lead to various physiological and psychological conditions, accelerate the development of burnout, and negatively impact well-being in the workplace [104]. Again, The Job Demands-Resources (JD-R) model posits that persistent job demands deplete employees’ mental and physical resources, ultimately resulting in health issues, including burnout [105,106]. Research has demonstrated that elevated quantitative and emotional job demands significantly contribute to higher levels of burnout and exhaustion among employees [107,108]. Continuous exposure to stress and challenging work conditions exacerbates these effects, leading to severe health impairments [109,110,111]. At this juncture, within the context of digital transformation in the field of ICT, there is an increasing use of technology in business processes. This involves continuous communication with customers and managers through email and other electronic communication tools in the workplace, as well as the pressure to quickly respond to or solve incoming requests (telepressure). Additionally, there is an increase in workload (techno-overload), exceeding the boundaries between work and personal life (techno-invasion), and the technostress resulting from high job demands. Alongside the ease of work processes facilitated by technological advancements, the inadequacy of resources provided to bank employees in the work environment is leading to a depletion of their resources, potentially resulting in burnout in the medium and long term. In a study conducted on 245 bank employees, it was found that technostress negatively affects the work–life balance of employees and leads to burnout [52]. Again, a study conducted in Spain involving 1037 bank employees revealed that technostress affects emotional exhaustion, an essential dimension of burnout [9]. In addition, a study conducted on bank employees in Indonesia empirically revealed that technostress reduces job satisfaction and that burnout significantly affects this issue [23]. According to the study results (H1), technostress experienced by bank employees influences their levels of burnout. This finding is consistent with the existing literature. Furthermore, the study’s results empirically highlight the need to emphasize this issue, particularly in rapidly digitalizing sectors such as the banking industry.

The study also found that musculoskeletal disorders (MSDs) play a moderating role in technostress’s impact on bank employees’ burnout levels. Specifically, MSDs significantly amplify the effects of technostress on burnout and raise the incidence of burnout among bank employees with MSDs. Work-related stress experiences of employees cause the development of somatic symptoms, and, together with pain, reduce the employee’s perception and strengths regarding demands related to the job; the employee’s resources become depleted, and they experience burnout [112,113]. Many studies are in the literature on the relationship between job-related stress and the development of MSDs [114,115,116]. In a longitudinal study conducted on MSDs and job-related stress, it is emphasized that the direct relationship between the two cannot be solely explained by the effect of stress on MSDs. It is suggested that there may be other factors contributing to the experience of MSDs, and the findings regarding a causal direction are not yet clear. It is also noted that more research is needed in this area. Additionally, it is highlighted that employees with high MSDs may have a higher perception of stress [113]. From this point of view, MSD status was examined holistically and as a moderator variable in the study. When the extant literature was discussed, it was determined that the relationship between stress levels and burnout perceptions of 161 diplomatic employees was higher in MSD bank employees than in those without an MSD [75]. In addition, a study conducted on nurses found a significant relationship between high job demand, job stress, and MSD, which was associated with burnout at a correlational level [117]. In a study conducted on healthcare college students, it was found that those who experienced high levels of burnout also had musculoskeletal disorders (MSDs) [118]. A study conducted on healthcare workers revealed an effect between stress and burnout, sleep quality, and MSDs [119]. A survey of German childcare workers found that excessive commitment to work and perception of low control over processes lead to musculoskeletal disorders [120]. When all of these studies are evaluated together, the results of the current study support and expand the existing literature, specifically within the banking sector.

## 5. Conclusions

Technological advancements and digital transformation have significantly changed the way businesses and individuals conduct their daily activities compared to the past. The development of information and communication technology (ICT) has led to high-performance business processes and increased competition, resulting in a greater reliance on technology and its impact on our lives. Sectors such as banking have rapidly embraced digitalization, and the shift to remote work flexibility following the pandemic has made the use of ICT a necessity rather than a choice for many businesses. While the use of ICT has brought about significant improvements in performance and efficiency, it has also led to challenges such as job-related stress, known as technostress, for employees. As empirically demonstrated by the results obtained within the scope of the study, technostress experienced by employees causes employees to experience resource depletion and burnout in the medium and long term. Again, technostress contributes as a factor, and the MSD experiences of employees in the work environment further strengthen this negative interaction mechanism.

It is important to highlight that neither MSDs nor burnout are desirable outcomes. The study specifically examined the relationship between technostress, MSDs, and burnout and how these factors influence each other in the workplace. Implementing proper workplace organization is a proactive measure that can help reduce these issues. At this point, within the framework of a sustainable management approach, it would be appropriate to take essential steps to solve the physical and psychological reflections of technostress, especially MSDs and burnout. Implementing realistic and timely policies to reduce technostress as a positive job source, especially in the workplace, is essential for maintaining high efficiency, employee well-being, and a positive organizational climate achieved through ICT and digitalization.

In conclusion, although ICT and digitalization offer numerous advantages, organizations must be mindful of the potential negative consequences on employee health and well-being. By addressing technostress and its related outcomes through thoughtful workplace organization and supportive policies, businesses can strive to create a balanced environment that maximizes the benefits of technology while minimizing its drawbacks.

## 6. Theoretical and Practical Implications

This study provides theoretical and practical insights for researchers and professionals in the rapidly digitalizing sectors for effective management in the banking sector. As theoretical insights, the study first expands the literature on ICT-induced work-related stress by investigating the impact of technostress and burnout. In the extant literature, the ongoing debate regarding the effects of technostress on burnout remains unresolved in the academic literature [25,49]. Consequently, the direct relationship between using information and communication technology (ICT) and burnout is still ambiguous. The current body of evidence does not link these two variables definitively [50]. Although various studies have explored different facets of this relationship, the findings have often been inconsistent, indicating the need for further research to clarify this complex interaction. Therefore, understanding how ICT usage contributes to or mitigates burnout continues to be an important area of investigation for researchers and practitioners alike. Once again, there are discussions about the interaction between technostress and burnout. These discussions either generalize the issue by including various employees or focus mainly on healthcare and IT workers [51]. There are also calls to address this issue in more detail within different sectors and professions [52]. The prevalence of technology in different sectors and its impact on employees in the service process may result in varying levels of technostress and its manifestations. A study focusing on the banking sector aimed to develop a scale for measuring technostress and found that the level of technostress was relatively low. The study emphasized the importance of providing evidence to examine the situation of technostress and its potential outcomes, specifically within the banking sector, which would significantly contribute to the existing literature [53]. Considering these calls, the current study expands the literature by empirically examining the relationship between technostress and burnout in the banking sector. Secondly, it emphasizes that the literature has significant deficiencies in using moderator variables for the effects of technostress and ICT-induced stress [72]. It is shared that the moderators generally used are variables closely related to technology and IT (such as technical support, literacy facilitation, and involvement facilitation). However, there is a lack of research that explores techniques and interventions to alleviate technostress and provides recommendations and awareness [31,73,74]. There are also calls for a more nuanced examination of the relationship between stress, burnout, and MSDs. This could involve looking at specific types of stress, such as technostress within particular occupational groups, and delving into the mechanisms of this intricate interaction [62,75,76]. A study conducted specifically for the IT sector determined that job demands such as long working hours, overtime work, forced posture, and occupational stress have a very high relationship with MSDs [121]. Furthermore, it is essential to note that office workers who sit for long periods (more than 4 hours per day) are at a high risk of developing musculoskeletal disorders (MSDs) [122]. Based on these points, the relationship between technostress, burnout, and MSDs was examined empirically in detail, and the literature was expanded to respond to the calls in the literature.

The study expands the literature on the transactional and person–environment fit approaches. It states that there is harmony and interaction between the individual and their environment and that constant stress or misfit between the employee and the work environment can lead to adverse physiological and psychological effects. The study empirically demonstrated that when employees face increasing workloads due to technological elements and a misfit between work environment expectations and their own, they will experience burnout. The COR and J D-R theories are further developed by empirically testing the interaction between technostress and burnout. A study focused on bank employees clearly demonstrates that technostress, induced by high job demands such as technological overload from ICT opportunities and digitalization, as well as technological invasion where work–life boundaries are exceeded by the expectation of constant availability and fast response, leads to employee burnout by depleting their resources.

The study provides valuable insights and practical implications for organizations, especially in the finance and banking sectors, where digitalization is indispensable, to establish a healthy workplace management practice. Technostress elements such as techno-overload and techno-invasion, created by high job demands originating from technology in the business environment, cause employees in the banking sector to experience burnout. This issue should be urgently addressed regarding sustainable management approaches and public health, especially in the locomotive sectors of digital transformation such as finance, banking, telecommunications, etc. Indeed, the continuity of performance increases provided by an increase in efficiency and productivity is based on the well-being of the existing employees and the interaction of a healthy work environment. Technostress is a unique form of stress that is caused by the use of digital technology and ICT in the workplace. Unlike other stress factors, it cannot be easily eliminated and instead requires ongoing management. When dealing with technostress, businesses should prioritize their employees’ well-being over just focusing on performance and efficiency gains. Although some may view workplace stress as a driver of high performance and creativity, it is important to note that this is not always the case. The assumption that heightened arousal leads to increased performance, known as the Yerkes–Dodson Law, needs to be theoretically tested. Research suggests that this assumption is flawed and that long-term stress can lead to decreased performance at the individual and organizational levels [123]. The long-term autonomic arousal experienced by employees can negatively impact their health by leading to conditions such as heart disease, mental and physical depression, burnout, and anxiety [123,124,125,126]. Therefore, work-related stress factors, particularly technostress, need to be emphasized. While technological advancements and the use of technology are essential for performance and efficiency focused on customer satisfaction, it is important to take steps to reduce the adverse effects on employees. The literature emphasizes that increasing employee awareness and developing self-control towards events and situations can help prevent adverse outcomes such as burnout [127,128]. At this point, first of all, it would be appropriate to determine a policy to take measures to increase employee mindfulness and workplace buoyancy. Workplace buoyancy stands out as an essential personal resource that employees can develop within the scope of JD-R regarding the current and future potential difficulties they experience at work. In work environments that require high job demands, workplace buoyancy stands out as an essential personal resource in reducing employees’ stress and burnout experiences that may occur due to this [129]. Again, mindfulness is a dynamic state of conscious awareness in which individuals are implicitly aware of the context and content of information, leading to heightened involvement and alertness [130,131,132]. The core concept of mindfulness is to remain present in the moment rather than dwelling on past experiences or anticipating future events. Mindfulness, which is approached through various perspectives, particularly trait mindfulness (also known as dispositional mindfulness), represents a flexible understanding that can be developed through training and intervention programs rather than as a static construct [133,134]. Dispositional mindfulness refers to a consistent, trait-like quality that influences how individuals perceive and interact with the world around them. This quality can be developed through a structured training program incorporating practices such as meditation, mindful breathing, and mindful movement. By engaging in these practices, individuals can enhance their awareness of their thoughts, emotions, and interactions with the environment. This increased awareness allows people to respond to situations more thoughtfully and deliberately rather than reacting automatically. As a result, individuals who cultivate dispositional mindfulness often experience improved emotional regulation, reduced stress, and enhanced overall well-being. Research indicates that higher levels of dispositional mindfulness are associated with numerous psychological benefits, such as reduced work-related stress, improved emotional regulation, and enhanced well-being [135,136,137,138]. Research in the literature strongly emphasizes that practicing mindfulness can help reduce technostress [3,98]. In this context, the human resources department and senior management can implement an effective employee training program by utilizing internal and external resources to enhance their mindfulness and workplace resilience. This approach can provide bank employees with the necessary talent and personal resources to disrupt the negative interaction mechanism created by technostress, which often leads to burnout. Secondly, employees should be given the opportunity and tools to engage in digital detox practices. Digital detox refers to a designated period during which individuals intentionally avoid using electronic devices, including smartphones. This practice aims to help reduce stress and enhance one’s focus on face-to-face social interactions and engagements in the physical world. By temporarily disconnecting from the digital realm, people can experience a range of benefits, such as improved mental and psychological well-being and reduced stress, depression and anxiety [139,140], and cortisol levels [141,142,143,144]. It also reduces the cognitive overload experienced by employees within the scope of technostress [145]. Implementing digital detox strategies in the workplace can enhance employee well-being and productivity. Critical approaches include scheduling regular breaks, establishing “no screen” hours, and creating tech-free zones [146]. Promoting mindfulness through meditation and educational workshops on digital detoxing and digital well-being seminars are also effective methods. Thirdly, administrative support plays a primary role in mitigating individuals’ technostress experiences [34]. The human resources department should provide awareness training to managers at all levels about technostress. A training program should be organized to support employees and demonstrate the correct approach through various methods. Additionally, managers should implement the digital detox buddy system and ensure its establishment. Leaders should promote digital detoxing by encouraging team members to pair up with colleagues for mutual support and accountability. This collaborative approach makes the process more enjoyable and engaging, turning it into a fun challenge rather than a burdensome task [147]. Additionally, implementing leadership development programs to enhance managers’ leadership styles can help decrease employee technostress and burnout. Research shows that leadership qualities such as transformational, empowering, and digital are associated with significantly reducing technostress in employees [14,148,149,150]. At this point, it is essential to organize leadership-development training programs for current managers. This will help prepare them to implement a leadership style that is adept at change management, has a vision for digital applications and reflections, possesses excellent communication skills, embraces open-door communication, and closely engages with subordinates, fostering high leader–member interaction. By doing so, employees’ experiences of technostress due to high job demands can be mitigated, ultimately reducing the burnout effect. Additionally, it can enhance administrative support and leader–member exchange (LMX) as a positive resource in the organizational environment, reducing employees’ negative experiences [128].

Another important practical implication of the study is that individuals with MSDs (musculoskeletal disorders) are more likely to experience burnout due to technostress. This awareness is essential for businesses. The study highlights the relationship between work-related stress, somatic effects, and MSDs, emphasizing that individuals with high MSDs are more likely to experience high levels of burnout. A comprehensive systematic review study suggests that implementing resistance training, stretching programs, and improving ergonomic conditions in the workplace can significantly contribute to preventing MSDs [151]. The human resources department can substantially contribute to solving the problem by developing musculoskeletal disorder (MSD) policies to combat MSDs. A study has also emphasized that the number of businesses with MSD policies is low, and this challenge can be significantly overcome through determined decision-making and workplace practices [152]. A program can be designed with a specialist physiotherapist. The program aims to raise awareness of musculoskeletal disorders (MSDs) and provide training on how to eliminate harmful practices. Additionally, it can include more comprehensive and personalized follow-ups, such as scheduled stretching sessions with a physiotherapist and occupational therapist, to address the factors contributing to MSD development. The presence of employees specializing in organizational psychology is crucial for accurately assessing psychological workplace issues. Similarly, having a physiotherapist to enhance physical health or provide occupational therapy can significantly benefit human resources practices and ensure sustainable employee health and satisfaction. A multimodal physiotherapy program and workplace exercises supported by a work-focused approach are emphasized as being highly effective in combating MSDs, particularly in preventing workplace-related MSDs [153,154,155]. Multimodal treatment refers to a therapeutic approach that integrates multiple types of treatments within a single session. When managing whiplash, this approach can encompass a wide range of interventions. These may include active movements to promote mobility, strengthening exercises to build muscle support, and muscle re-education to restore proper function. Additionally, kinesthetic exercises can help improve awareness of body movements, while posture correction addresses alignment issues. Functional exercises enhance daily activities, and manual therapies such as manipulation, mobilization, and massage can relieve pain and improve movement. Electrotherapy may be used to stimulate muscles and reduce pain, while professional advice and education empower patients with knowledge about their condition. A home exercise program can encourage ongoing rehabilitation outside of clinical settings. In some cases, medication may be prescribed to manage symptoms, and a soft collar might be used for additional support. Essentially, multimodal treatment combines several methods to provide a comprehensive approach to managing whiplash. Again, manager support is essential in the effective implementation of MSD policy. The European Agency for Safety and Health at Work, which offers preventive measures to reduce the effects of MSDs at individual and organizational levels, points out that excessive job demands should be controlled by the proper operation of the worker support system by managers in the formation of MSDs caused by psychosocial factors, and that the psychosocial catalysts of MSDs can be eliminated with an effective communication mechanism [156]. In this context, it would be appropriate to train managers to implement the worker support system and develop policies in the enterprise to encourage good practices. It is also emphasized that MSD risk prevention is not implemented in many enterprises, which creates performance, quality, and many adverse workplace outcomes while jeopardizing the well-being of employees. At this point, the human resources department should review its practices regarding MSD risk management and increase awareness of MSD risk management among senior management and employees.

## 7. Limitations and Future Directions

Despite the valuable insights provided by this study, it is important to acknowledge its limitations, which open up significant avenues for future research. One primary limitation is the cross-sectional design, which inherently restricts the ability to draw causal inferences. Future research could benefit greatly from longitudinal and experimental designs that examine the current model over time, thereby enhancing the capacity to establish causality. Secondly, the study’s sample size was relatively small, limiting the findings’ generalizability. Future research could explore whether these results apply to a broader range of organizations and cultural contexts. By employing larger and more diverse samples, researchers could gain deeper insights into the impact of technology on burnout and MSDs. Such studies would help to strengthen the evidence base and provide more robust conclusions regarding these psychological and ICT-induced stress phenomena. The extant literature emphasizes that the gender factor has a decisive effect, especially in the MSD experience [62,157]. Studies have shown that MSDs in women are higher than in men. Examining this situation, especially among bank employees, and testing it in other sectors may provide an essential perspective on overcoming the negative interaction between technostress, MSDs, and burnout. Furthermore, as highlighted in previous studies [25,158], it would be beneficial for future research to consider age and tenure when examining the impact of technology use on technostress and musculoskeletal disorders (MSDs). This approach could help identify high-risk groups and significantly contribute to the field. Moreover, conducting research to explore the relationship between technostress, musculoskeletal disorder (MSD) components, diaphragm, abdomen, posture, respiratory functions that may affect MSDs, and burnout in the banking sector or other professions and industries with high ICT use could provide valuable insights for both academic literature and practical applications. These studies could raise awareness about specific body parts requiring targeted training and exercise.

## Figures and Tables

**Table 1:** Measurement model analysis results.

Construct/ Items		Factor Loadings	Mean	Cronbach’s Alpha α ≥ 0.70	Composite Reliability α ≥ 0.70	AVE α ≥ 0.50	VIF
Burnout	Br1	0.894	3.177	0.899	0.901	0.771	3.757
	Br2	0.928					4.049
	Br3	0.779					1.765
	Br4	0.904					3.318
Techno-invasion	İnv1	0.814	3.079	0.861	0.863	0.706	1.723
	İnv2	0.852					2.258
	İnv3	0.870					2.427
	İnv4	0.824					1.957
Techno-overload	Tov1	0.712	3.036	0.847	0.874	0.687	1.527
	Tov2	0.865					2.535
	Tov3	0.877					2.131
	Tov5	0.850					2.270
Technostress 2nd Order Construct	Techno-invasion	0.924	3.097	0.789	0.805	0.825	1.740
	Techno-overload	0.892					1.740

**Table 2:** Fornell–Larcker criterion matrix.

		1	2	3
1	Burnout	0.878		
2	Techno-invasion	0.405	0.840	
3	Techno-overload	0.342	0.652	0.829

**Table 3:** Heterotrait–Monotrait ratio (HTMT) matrix.

		1	2	3
1	Burnout			
2	Techno-invasion	0.457		
3	Techno-overload	0.384	0.762	

**Table 4:** Heterotrait–Monotrait ratio (HTMT) matrix.

	Burnout
Burnout	
Technostress	0.488

**Table 5:** Fornell–Larcker criterion matrix.

	Burnout	Technostress
Burnout	0.878	
Technostress	0.413	0.908

**Table 6:** MGA analysis results.

Pathways	β	t Value	*p* Value
Technostress -> Burnout (MSD-0)	0.283	3.084	0.002
Technostress -> Burnout (MSD-1)	0.518	6.880	0.000
	Difference (msd0–msd1)	t value (|msd0 vs. msd1|)	*p* value (msd0 vs. msd1)
Technostress -> Burnout Difference (MSD0–MSD1)	−0.235	1.989	0.049

## Data Availability

The data supporting this study’s findings are available from the corresponding author upon reasonable request.
